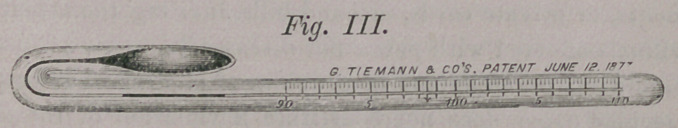# A Model Pocket Case

**Published:** 1878-04

**Authors:** Charles C. F. Gay

**Affiliations:** Surgeon to the Buffalo General Hospital


ART. IV.—*Description of a Model Pocket Case of Instruments.* By Charles C. F. Gay, M. D.,, Surgeon to the Buffalo General Hospital.

I wish to call attention to a Pocket or Dressing Case of Instruments which possesses merit enough, I think, to warrant a brief description for publication in the columns of the *Journal.* The average pocket case contains instruments, too many of which are useless, and is therefore too large to be conveniently portable. The pockets of physicians are usually encumbered, not only with the ordinary surgical case of instruments, but it is found necessary to carry a thermometer and hypodermic syringe. I have succeeded—through the co-operation of Geo. Tiemann & Co., Instrument Makers, New York,—in devising a case which accommodates all the instruments required, including both the hypodermic syringe and thermometer; the size of which is so small that if its length be lessened by three fourths of an inch, it will occupy in the pocket, no more room than the old fashioned hypodermic syringe case. Indeed it is so compact and small that it may be carried conveniently in the watch or vest pocket.

It contains two double—shell-handle—knives, one of which has a scalpel in one end and a probe-pointed bistoury in the other end, the blades are fastened open by a spring catch; the other handle has in one end an universal or tenotomy blade, and in the other end a scarificator or gum lancet, both blades fastened open by a

*Fig. I.*

spring catch—a pair of scissors (well represented in Fig. 1) with handles open in front to allow them to pass over or around adjoining instruments in order to economize space; one pair of artery forceps—a female silver catheter with caustic holder—a grooved director, one end of which can be bent and threaded to serve the purpose of an aneurism needle—a probe-pointed director, tongue spatula and wire twister—an exploring needle—a Hypodermic Syringe and Thermometer, both encased in metal.

*Fig. II.*

(Fig. II) represents the Syringe opened, ready for use, and also represents it closed. When closed the needle is taken off, reversed and enclosed in a metal case, which is screwed over it upon the Syringe proper.

*Fig. III.*

The Thermometer (Fig. III) is protected by a metal case, one end of which is closed and the other free or open, and is lined with flannel. The instrument need not be removed from its case until after the temperature is taken, and is therefore better protected from danger of breaking than any heretofore in use.

The case itself is made of Turkey Morocco, lined with silk and velvet; it has a pocket for needles and thread. It is four inches long by one inch and seven eights wide and seven eights of an inch thick, fastened by a sterling silver lock. It contains all the instruments that the physician or surgeon need carry about his person, and just those instruments that are most often needed, and indeed, which are continuously in every day use.

## Figures and Tables

**Fig. I. f1:**
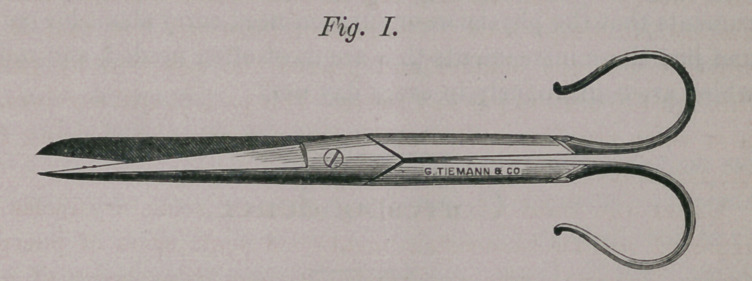


**Fig. II. f2:**
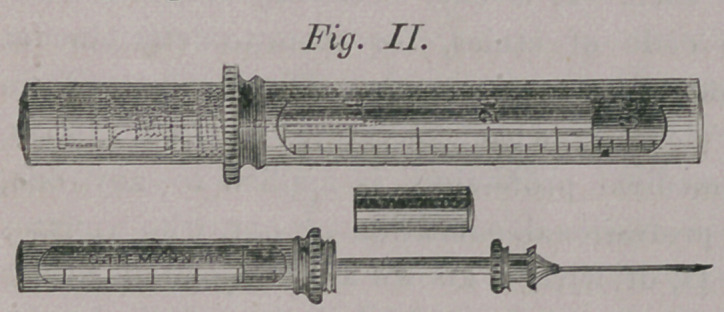


**Fig. III. f3:**